# Oxidative C-H activation of amines using protuberant lychee-like goethite

**DOI:** 10.1038/s41598-018-20246-y

**Published:** 2018-01-31

**Authors:** Sanny Verma, R. B. Nasir Baig, Mallikarjuna N. Nadagouda, Rajender S. Varma

**Affiliations:** 1 Oak Ridge Institute for Science and Education, P. O. Box 117, Oak Ridge, TN 37831 USA; 2 Water Systems Division, Water Resources Recovery Branch, National Risk Management Research Laboratory, U. S. Environmental Protection Agency, 26 West Martin Luther King Drive, MS 443, Cincinnati, Ohio 45268 USA

## Abstract

Goethite with protuberant lychee morphology has been synthesized that accomplishes C-H activation of *N*-methylanilines to generate α-aminonitriles; the catalyst takes oxygen from air and uses it as a co-oxidant in the process.

## Introduction

Inspired by nature, we aspired to design a protocol for the C-H activation of amines using one of the most benign^1^ and abundant metals in the earth’s crust, iron^2–9^. The ultimate success of this chemistry lies in the use of atmospheric oxygen in air as a replacement for the hazardous oxidants which include hydrogen peroxide and organic peracids^10–16^. In spite of burgeoning interest in this area, there are few reports available on this topic^17,18^; Murahashi group utilized molecular oxygen as an oxidant in the presence of ruthenium salt^17^ whereas the Fu and co-workers used a catalytic amount of radical initiator (Azobisisobutyronitrile) AIBN^18^. Homogenous vanadium-^19^ and iron-based catalysts^20,21^ were also reported for the same reaction. Recently, a heterogeneous ruthenium-based photocatalyst was used for the synthesis of α-aminonitriles under visible light irradiation^22^. However, to date none of the reports demonstrate the use of atmospheric oxygen for this reaction. The use of earth-abundant base metals and eco-friendly oxidants define the economic aspects and environmental impacts that are directly linked to the reaction pathway, atom economy and waste generation^23–27^. All of these limitations could possibly be addressed by replacing often deployed transition metals with benign ferrites^28^ and typical noxious oxidants with air. Success in this domain will address the growing economic and environmental concerns associated with a chemical synthesis. In this article, we present the outcome of our extensive exploratory work and experimentation leading to the discovery of a special morphological version of ferrites. We also address the stoichiometric needs for them to perform the C-H activation of amines under simple aerial atmosphere.

## Synthesis and Characterization of Catalyst

**Figure 1:**
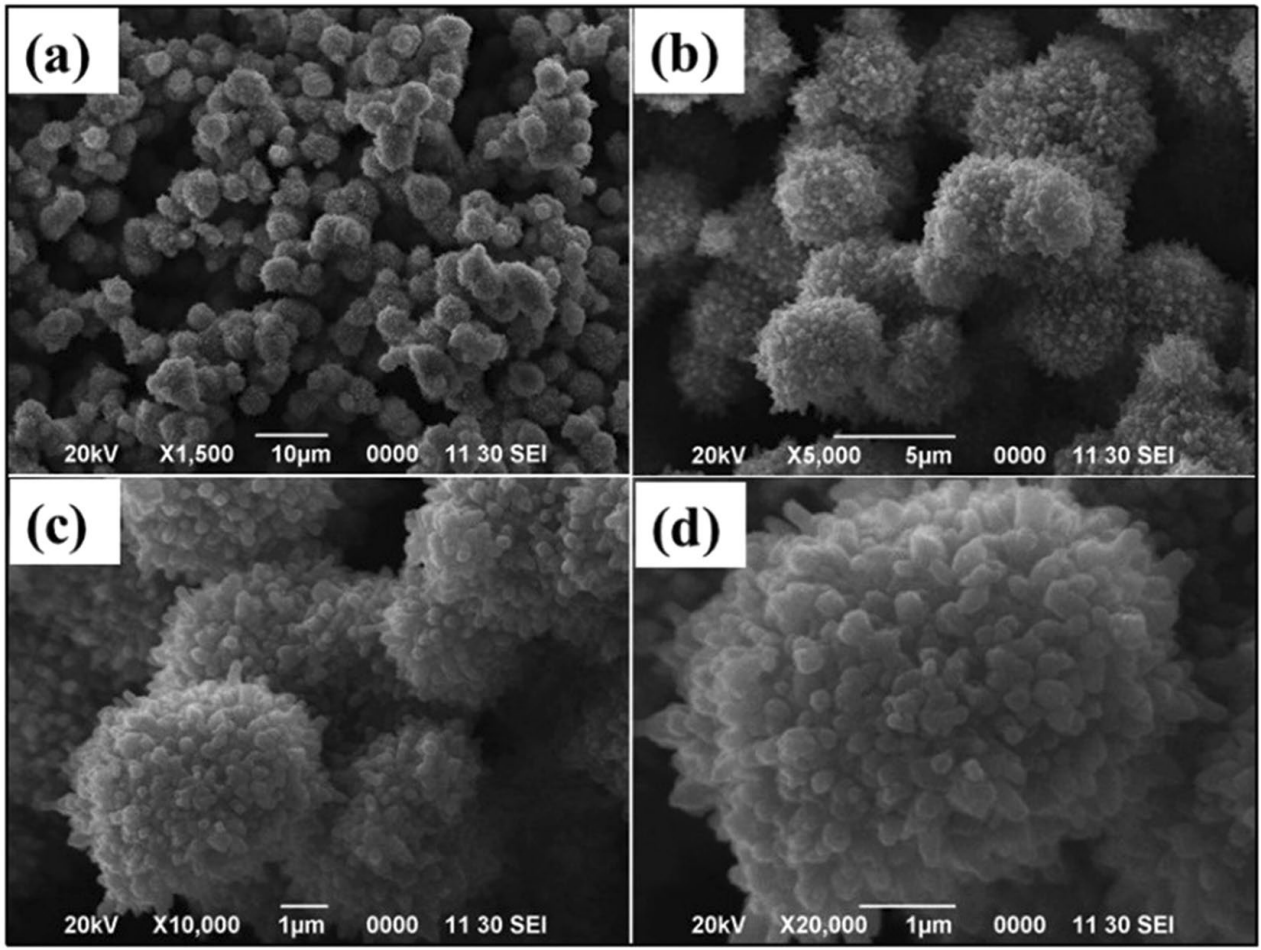
SEM images of PLG.

The First, commonly available iron salts and their oxide forms were screened for the C-H activation of amines. The objective was to find an iron salt that could convert amines to corresponding α-amino nitriles using air; *N, N*-dimethylaniline was chosen as a model substrate and the reactions were performed in aqueous media with assorted iron salts, namely Fe(OAc)_2_, FeCl_2_, FeSO_4_, FeCl_3_, Fe(NO_3_)_3,_ Fe_2_(SO_4_)_3_, Fe(acac)_2_, and Fe(acac)_3_ (Table 1, entries 1–8). None of these iron salts gave promising results; only traces of the product were detected even after 24 h of stirring at 50 °C (Table 1, entries 1–8). Next, some of the reported catalysts were screened (Table 1, entries 9–12). The reaction with FeO@GO (graphene supported iron oxide) under similar conditions gave 12% of desired product after 24 h whereas the use of pure iron ferrites (Fe_3_O_4_ and FeO) could not furnish more than 10% of the corresponding amino nitriles; iron supported on graphitic carbon nitride (Fe@g-C_3_N_4_) gave a 17% yield. Although ordinary iron salts and oxides did not succeed in this mission, we reasoned that the moderate success observed correlated to their morphological variations and crystalline nature thus increasing the oxygen absorption abilities and improving the catalytic activity for the desired C-H activation in the amino nitrile formation. At the outset, we embarked on the synthesis of iron oxides with varying morphologies and crystal pattern by treating the iron (III) salts with the amino acids. Several experiments were undertaken for the synthesis of iron oxide by heating the ferric sulfate with glycine, lysine, aspartic acid, asparagine, glutamine, and proline. Interestingly, we observed the formation of goethite with protuberant lychee-like morphology when reduced with proline (Fig. 1). In case of other amino acids as a reducing agent, we did not see any precipitation; the mother liquor remained clear even after 24 h of heating in an autoclave.

**Table 1:** Optimization of reaction conditions for C-H activation^a^.

Entry	Catalyst	Time (h)	Yield^b^
1	Fe(OAc)_2_	24	Trace
2	FeCl_2_	24	Trace
3	FeSO_4_	24	Trace
4	FeCl_3_	24	Trace
5	Fe(NO_3_)_2_	24	Trace
6	Fe_2_(SO_4_)_2_	24	Trace
7	Fe(acac)_2_	24	Trace
8	Fe(acac)_3_	24	Trace
9	FeO@GO	24	12%
10	Fe_3_O_4_	24	8%
11	FeO	24	6%
12	Fe@g-C_3_N_4_	24	17%
13	PLG	6	97%
14^c^	PLG	12	92%
15	Crushed PLG	24	5%
16	Goethite	24	8%
17	IO-1	24	5%
18	IO-2	24	5%
19	IO-3	24	9%
20	IO-4	24	4%
21	IO-5	24	6%
22	IO-6	24	4%
23	IO-7	24	4%
24	IO-8	24	5%

Reaction condition: (a) *N, N* -dimethylaniline (1.0 mmol), catalyst (10 mol%), NaCN (1.2 mmol), H_2_O (2.0 mL), air; (b) GC yield; (c) catalyst (5 mol%).

**Figure 2:**
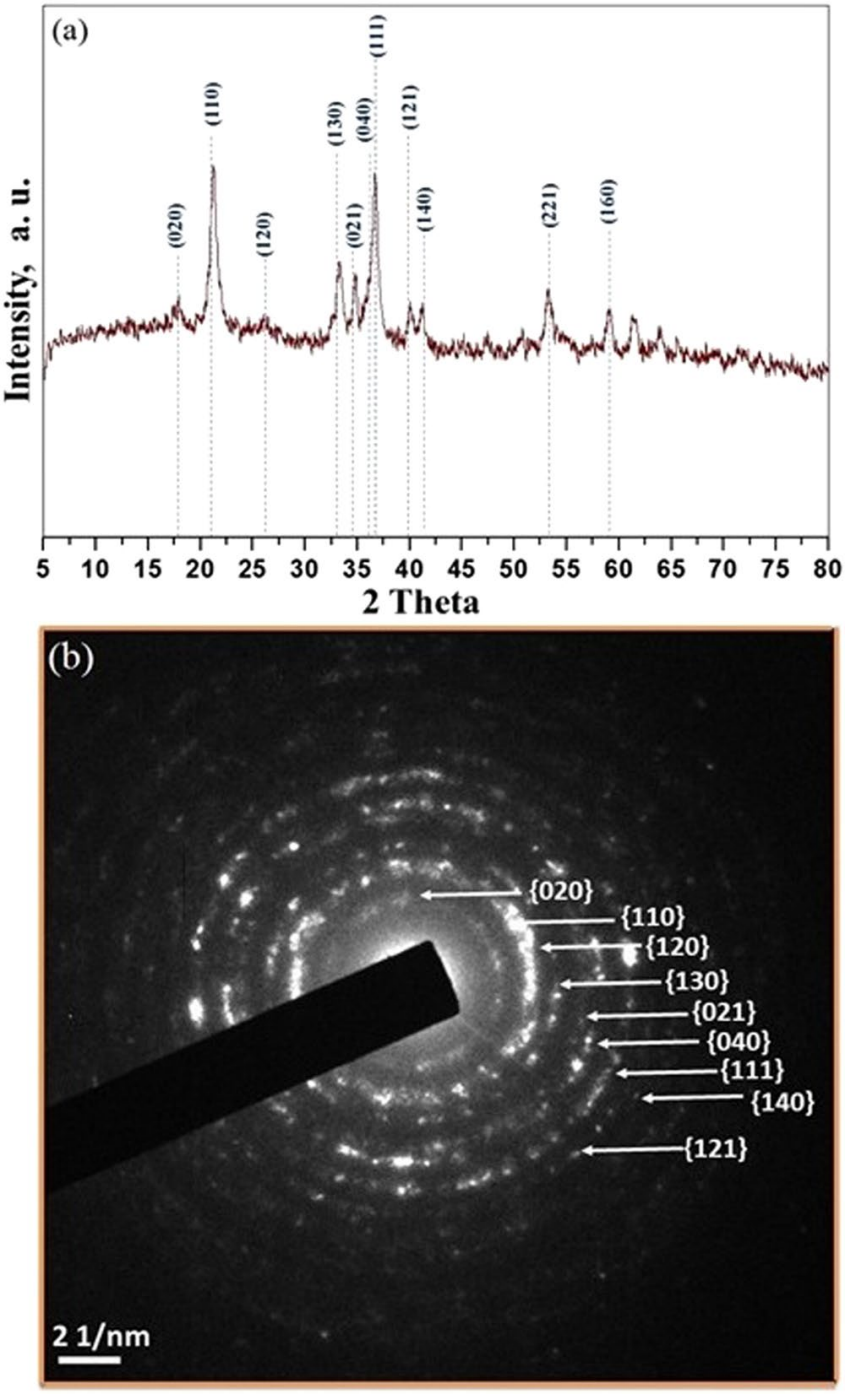
(**a**) XRD spectra and (**b**) SAED analysis of PLG.

**Figure 3:**
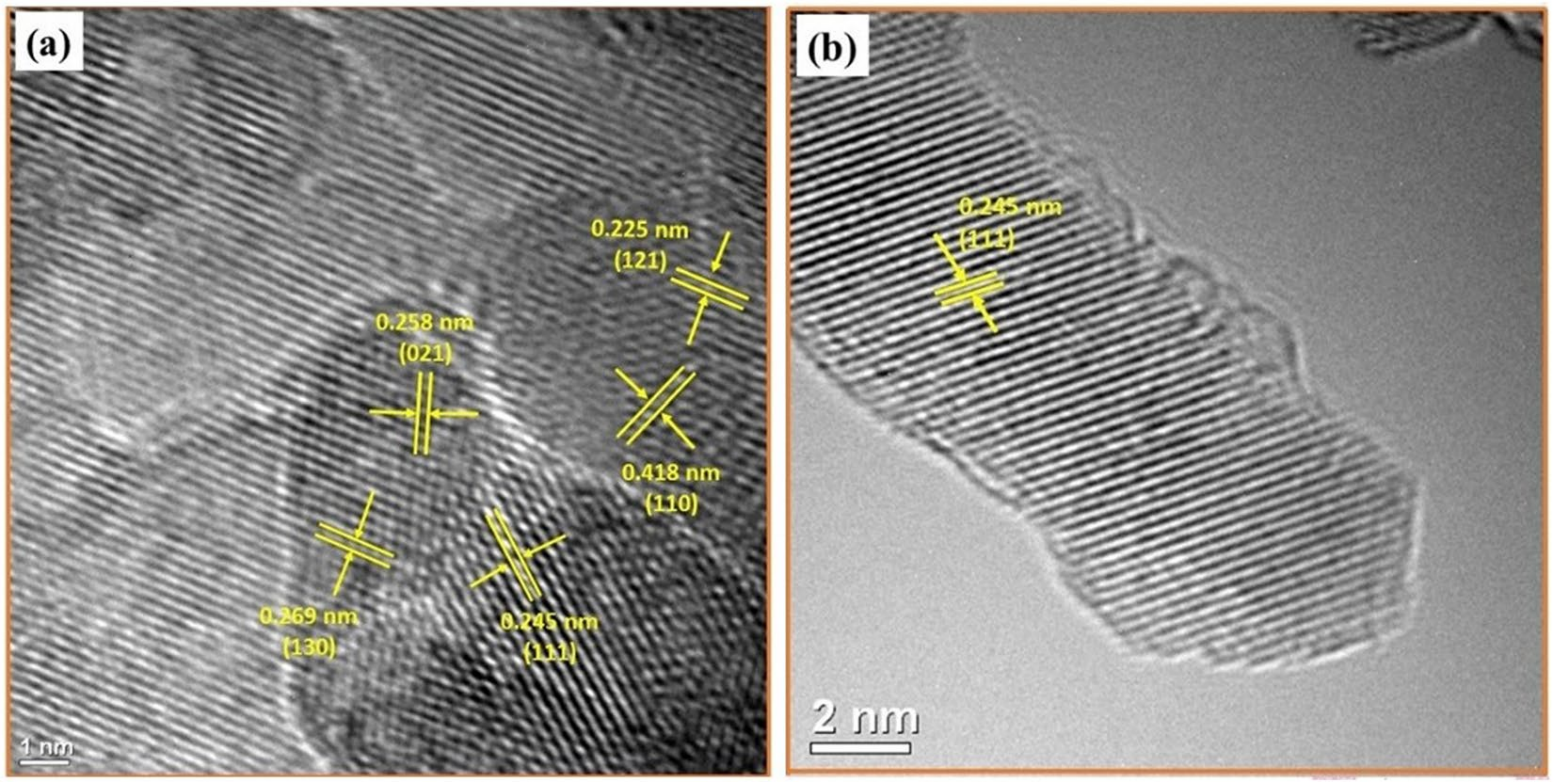
HRTEM images showing the lattice planes of PLG corresponding to the crystalline phase of goethite (JCPDS # 00-029-0713).

The morphology of synthesized goethite was obtained using scanning electron microscopy (SEM, FEI/Philips XL-30 Field Emission ESEM) and high-resolution transmission electron microscopy (HRTEM) obtained from JEOL 2010F microscopy. The elemental analysis was performed using energy-dispersive X-ray spectroscopy (EDX). X-ray diffraction (XRD) patterns were recorded on PANalytical X’Pert diffractometer using CuKα radiation (1.548 A°); the copper tube being operated at 45 kV and 40 mA. Scans were typically performed over a 2-theta range between 2 to 90°. XRD pattern analysis was performed using the MDI Jade XRD pattern processing computer software. X-ray photoelectron spectroscopy (XPS) was used to identify the oxidation states of iron. The XRD pattern of protuberant lychee goethite indicates the catalyst is enriched with crystalline nanoferrites (Fig. 2a). The observed peaks at 17.76°, 21.22°, 26.32°, 33.242°, 34.70°, 36.05°, 36.65°, 39.98°, 41.18°, 53.23°, and 59.16° are, respectively, representing the (020), (110), (120), (130), (021), (040), (111), (121), (140), (221), and (160) Bragg reflection as compared with JCPDS standards (00-029-0713) which confirmed the formation of Fe^+3^O(OH)^29,30^. Furthermore, selected area electron diffraction (SAED) analysis and HRTEM analysis were carried out to identify the crystalline phases of PLG (Figs 2b and 3) which support the XRD results. Further, the presence of iron is confirmed by EDX analysis (ESI, Figure S3) and of its oxidative state as iron (III) in PLG is confirmed by XPS analysis (ESI, Figure S4).

## Results and Discussion

**Figure 4:**
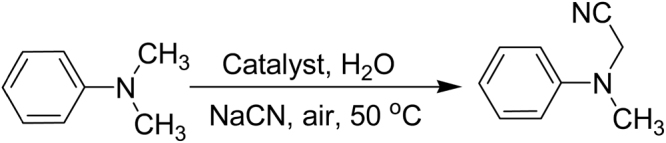
Synthesis of α-aminonitrile.

Goethite with lychee-like morphology was then screened for the synthesis of α-aminonitriles involving C-H activation (Fig. 4). The formation of corresponding α-aminonitriles became apparent within 6 h (Table 1, entry 13). The reduction in concentration of protuberant lychee goethite (PLG) from 10 mol% to 5 mol% led to the increase in required reaction time; 5 mol% of PLG took 12 h for the disappearance of starting material (Table 1, entry 14), whereas 10 mol% of PLG accomplished the task in less than 6 h (Table 1, entry 13). However, using the morphology-altered PLG material (by crushing PLG), gave only 5% of the desired product (Table 1, entry 15). Further, we tested commercially available different iron-based catalysts having similar chemical compositions but different morphologies for the synthesis of α-aminonitriles. Outcomes from these catalyst such as goethite (30–63% Fe), iron(III) oxide (≥99.995% trace metals basis; IO-1), iron(III) oxide hydrated (catalyst grade, 30–50 mesh; IO-2), iron(III) oxide powder (<5 μm, ≥99%; IO-3), iron(III) oxide nanopowder (<50 nm particle size; IO-4), iron(III) oxide puriss (≥97.0%, IO-5), Iron(III) oxide purified (≥95%; IO-6), iron(III) oxide (dispersion nanoparticles, ≤110 nm particle size, 15 wt.% in ethanol; IO-7) and iron(III) oxide (dispersion nanoparticles, ≤110 nm particle size, 20 wt.% in H_2_O; IO-8) are summarized in Table 1, (entries 16–24). However, these catalysts gave very low product yields. This observation reaffirms that protuberant morphology predicates the oxidative C-H activation under these moderate conditions.

After having the active catalyst in hand, it was imperative to establish its general reactivity. A wide range of *N*-methylaniline derivatives were subjected to α-aminonitrilation (Table 2, entries 1–7). Most of the reactions were completed in less than 6 h (Table 2). A substrate with free N-H group efficaciously afforded the formation of the corresponding α-aminonitrile in almost quantitative yield (95%). Substrates with electron withdrawing group (Table 2, entries 2–3) and electron donating group (Table 2, entries 5–7) did not show any peculiar effect.

**Table 2:** C-H activation of secondary and tertiary amines using protuberant lychee-like goethite^a^.

Entry	Substrate	Product	Yield^b^
1			93% (95%)^c^
2			88% (91%)^c^
3			87% (90%)^c^
4			94% (97%)^c^
5			92% (95%)^c^
6			94% (96%)^c^
7			92% (96%)^c^

Reaction condition: (a) Amines (1.0 mmol), PLG (10 mol%), NaCN (1.2 mmol), H_2_O (2.0 mL), air, 50 °C; (b) Isolated yield; (c) GC yield.

**Figure 5:**
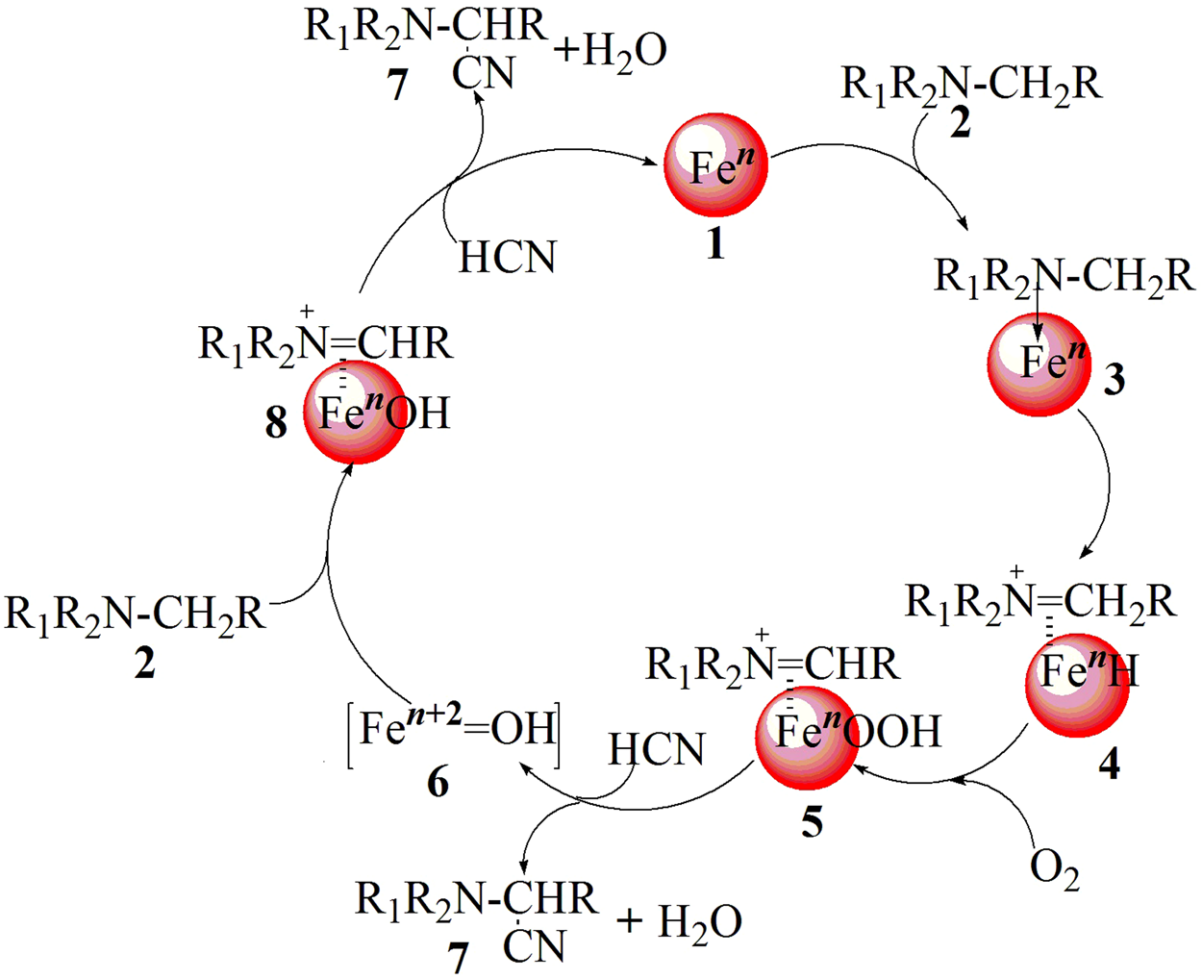
Plausible mechanism for the synthesis of α-aminonitriles.

This reaction proceeded *via* oxidative and reductive mechanism involving atmospheric oxygen (Fig. 4). At the first stage, iron species **1** coordinate with *N*-methylanilines **2** leading to the activation of C-H bond and creating an electron deficient iminium [iminium ion]-iron hydride complex intermediate **4**. The ensuing iron-hydride species **4** undergo reaction with molecular oxygen to form an [iminium ion]-Fe^*n*^OOH complex **5**. The nitrile ion (-CN) attacks the electron deficient carbon of the intermediate **5** which finally provides the formation of corresponding aminonitrile **7**, H_2_O and a Fe^*n*+2^ = O species, **6**. Thus, **6** reacts with another *N*-methylanilines, **2**, to give iminium ion intermediate **8** *via* an electron transfer and following hydrogen transfer. Cyanide reacts with **8** to afford α-aminonitrile, **7**, and Fe^*n*^, **1**, to complete the catalytic cycle (Fig. 5).

## Recycling of PLG catalyst for the C-H activation of ***N, N***-dimethylaniline

To check the recyclability of the PLG catalyst, a set of experiments were performed using *N, N*-dimethylaniline as a model substrate in water. After the completion of each reaction, the PLG catalyst was recovered *via* filtration, washed with water and reused for the oxidative cyanation of a fresh batch of *N, N*-dimethylaniline; PLG catalyst was reused six times (Supplementary Information). A significant decrease in product yields was obtained after the second run (Supplementary Information; Figure S1). Metal leaching in the reaction was examined using ICP-AES analysis wherein mother liquor did not confirm any traces of iron. Further, SEM analysis of recycled PLG was recorded up to six runs which showed a substantial change in its morphology (Supplementary Information; Figure S2–S6). This affirms that lychee-like morphology of catalyst plays an important role in activation of aerial oxygen during C-H activation.

## Conclusion

We have designed and developed a material which adsorbed oxygen from air and used it as a co-oxidant in C-H activation of *N*-methylanilines. The synthesized goethite with lychee-like morphology has proven its prowess as a very effective catalyst for an eco-friendly synthesis of α-amino nitriles by activating the C-H bond. Essentially, by using goethite, we can perform cyanation of *N*-methylanilines using atmospheric oxygen in aqueous media under neutral conditions.

## Electronic supplementary material

Supplementary information
