# Supplementary material for: Oxidative C-H activation of amines using protuberant lychee-like goethite
Source: Sci Rep. 2018 Jan 31;8:2024. doi: 10.1038/s41598-018-20246-y (PMC5792549; doi:10.1038/s41598-018-20246-y)
Supplement: Supplementary file 1 — Supplementary information [file 41598_2018_20246_MOESM1_ESM.pdf]

Supporting Information

Oxidative C-H activation of amines using protuberant lychee-like goethite

Sanny Verma[a†], R. B. Nasir Baig[a†], Mallikarjuna N. Nadagouda[b], Rajender S. Varma[b*]

[a]Oak Ridge Institute for Science and Education, P. O. Box 117, Oak Ridge TN, 37831, USA.

[b]Water Systems Division, Water Resources Recovery Branch, National Risk Management Research Laboratory, U. S. Environmental Protection Agency, 26 West Martin Luther King Drive, MS 443, Cincinnati, Ohio 45268, USA.

E-mail: varma.rajender@epa.gov

[†] Equal contribution

Contents

General procedure for the synthesis of protuberant lychee-like goethite

General procedure for α-cyanation of amines *via* C-H activation

Figure S1: Recycling of protuberant lychee-like goethite catalyst for α-cyanation of amines *via* C-H activation

Figure S2: SEM analysis of recycled protuberant lychee-like goethite after second run

Figure S3: SEM analysis of recycled protuberant lychee-like goethite after third run

Figure S4: SEM analysis of recycled protuberant lychee-like goethite after fourth run

Figure S5: SEM analysis of recycled protuberant lychee-like goethite after fifth run

Figure S6: SEM analysis of recycled protuberant lychee-like goethite after six run

Figure S7: XPS analysis of protuberant lychee-like goethite

Figure S8: EDX analysis of protuberant lychee-like goethite

^{1}H and ^{13}C NMR spectra of products

General procedure for the synthesis of protuberant lychee-like goethite

Ferric sulfate (1 mmol), proline (2 mmol) and water (40 ml) were charged in a pressure reactor. The mixture was heated in 110 °C in an oven for 24 hours. After 24 hours the reaction temperature was brought down to room temperature. The goethite with protuberant lychee morphology was isolated using centrifugation, washed with water followed by methanol and dried under vacuum at 50 °C. Catalyst characterization was performed using scanning electron microscope (SEM), high-resolution transmission electron microscope (HRTEM), X-ray diffraction (XRD) and X-ray photoelectron spectroscopy (XPS).

General procedure for α-cyanation of amines *via* C-H activation

A 25 mL side-armed round bottomed flask equipped with a magnetic stirring was charged with amine (1mmol), NaCN (1.2 mmol) and catalyst (10 mol %). 5 mL of water was added in the reaction mixture. The reaction mixture was heated at 50 °C by keeping vessel open. The progress of the reaction was monitored by checking TLC at regular interval of time. After completion of the reaction the product was isolated using ethyl acetate extraction, dried over sodium sulfate and purified using column chromatography and characterized.

Note: -OH, -NH_2 and -CHO group functionalized *N*, *N*-dimethylaniline derivatives (4-hydroxy *N*, *N*-dimethylaniline, 4-amino *N*, *N*-dimethylaniline, 4-formyl *N*, *N*-dimethylaniline) did not give any traces of product; similarly, no discernable product formation was observed in the case of aliphatic amines (*N*, *N*-diethylamine, *N*, *N*-dibutylamine) and cyclic *N*-arylamines (1-phenyl piperidine, 1-phenyl pyrrolidine).

Recycling of protuberant lychee-like goethite catalyst for α-cyanation of amines *via* C-H activation

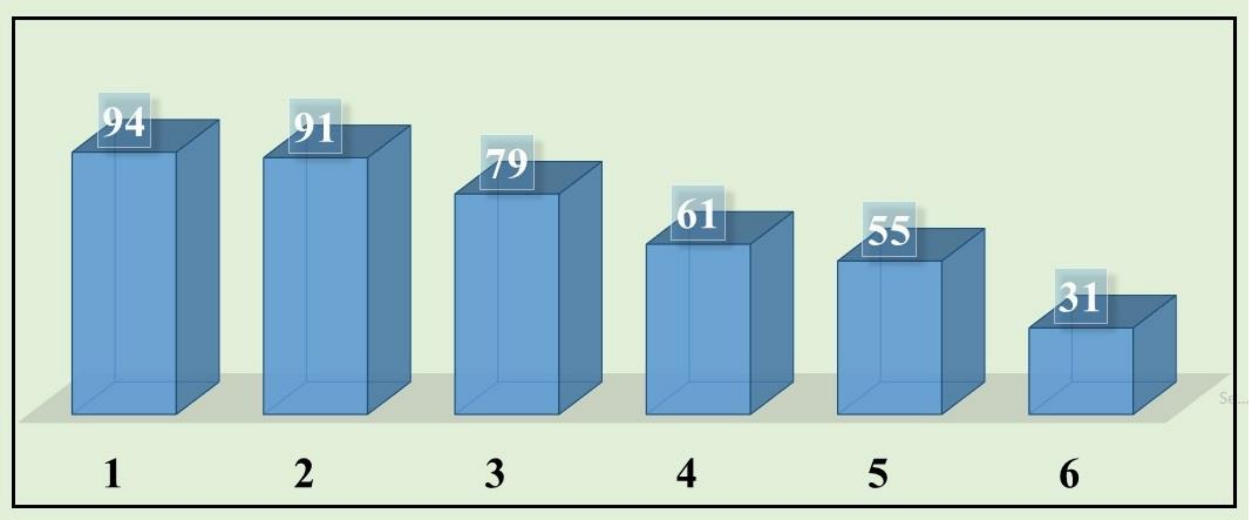

Figure S1. Recycling of protuberant lychee-like goethite catalyst for α-cyanation of amines *via* C-H activation

SEM analysis of recycled protuberant lychee-like goethite catalyst after second run

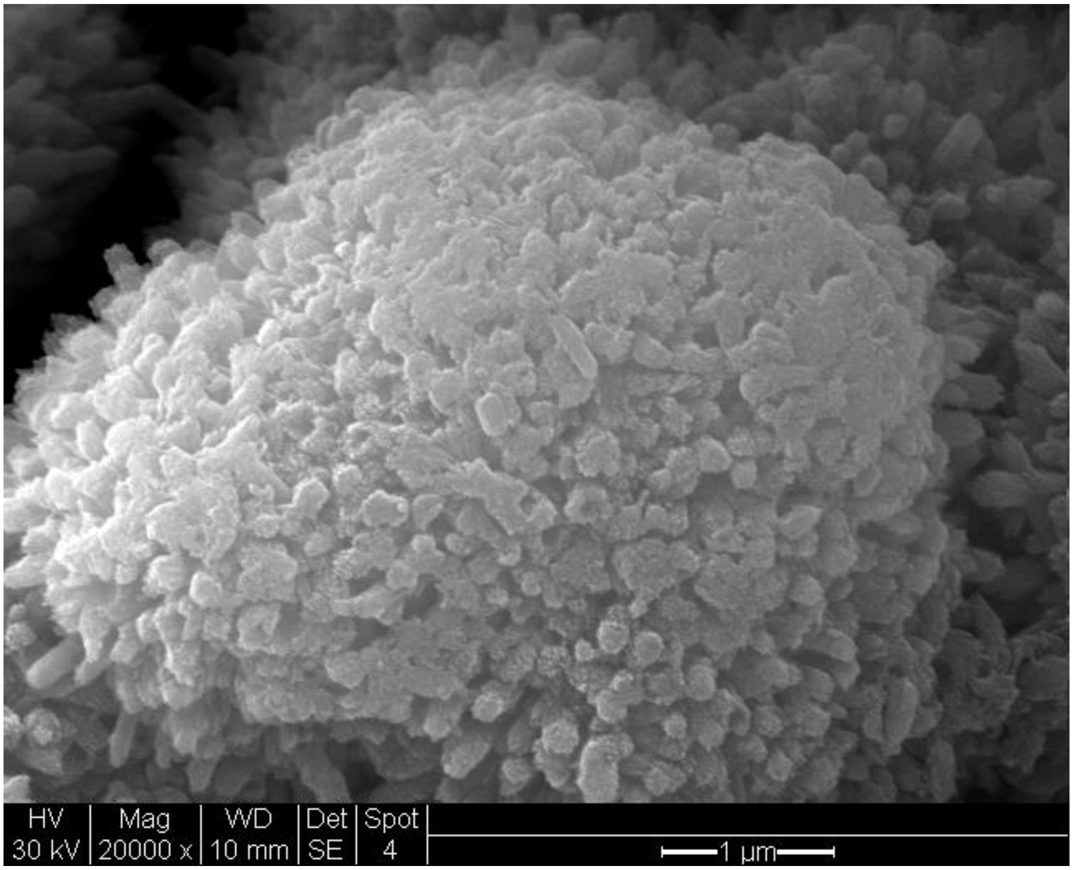

Figure S2. SEM analysis of recycled protuberant lychee-like goethite catalyst after second run

SEM analysis of recycled protuberant lychee-like goethite after third run

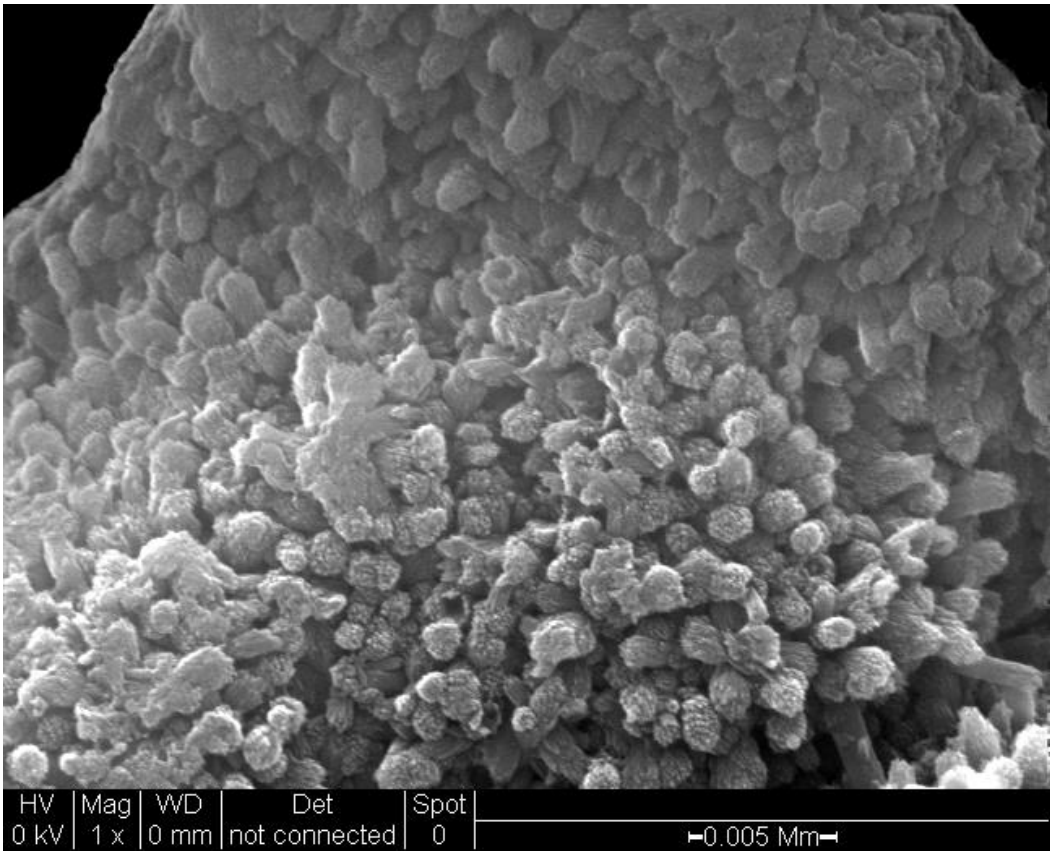

Figure S3. SEM analysis of recycled protuberant lychee-like goethite after third run

SEM analysis of recycled protuberant lychee-like goethite after fourth run

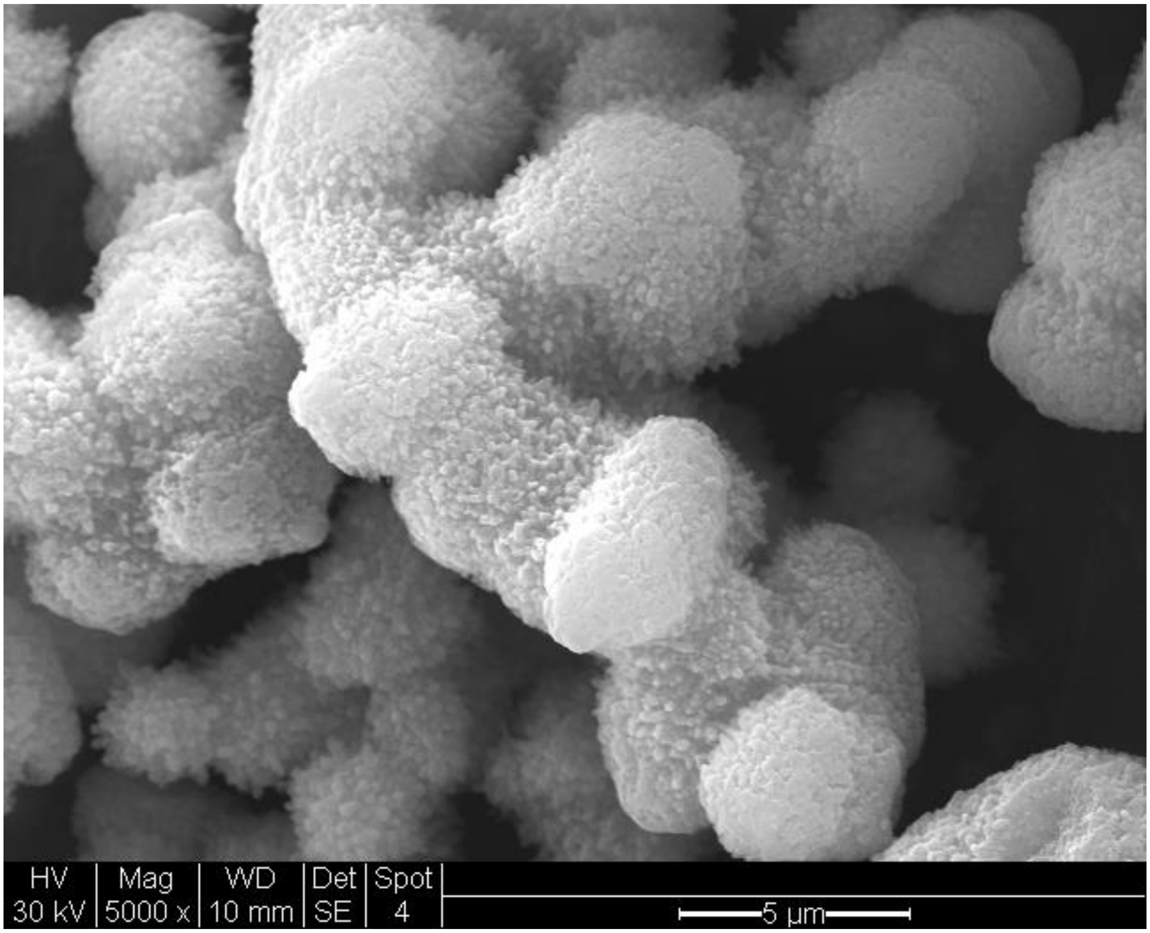

Figure S4. SEM analysis of recycled protuberant lychee-like goethite after fourth run

SEM analysis of recycled protuberant lychee-like goethite after fifth run

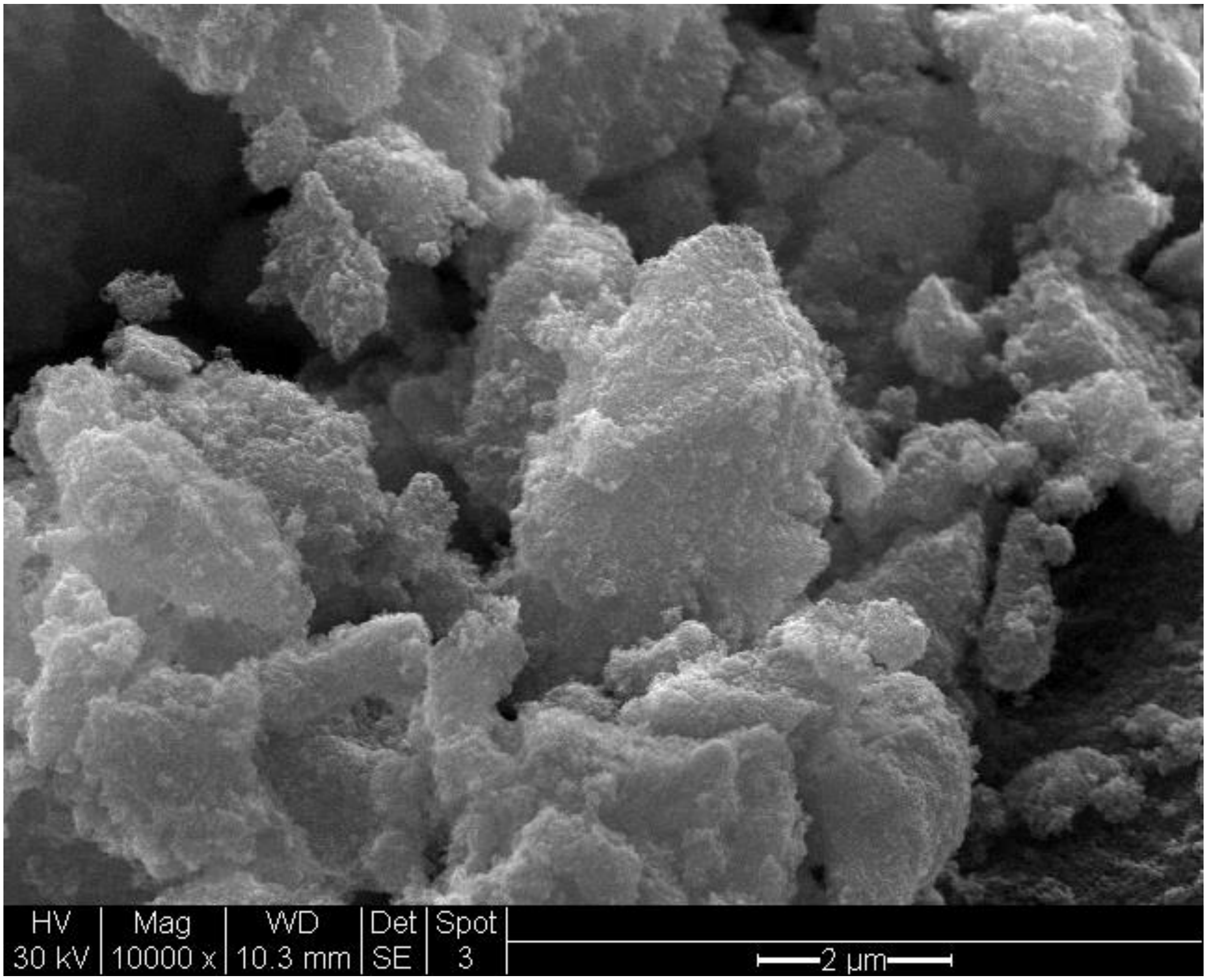

Figure S5. SEM analysis of recycled protuberant lychee-like goethite after fifth run

SEM analysis of recycled protuberant lychee-like goethite after sixth run

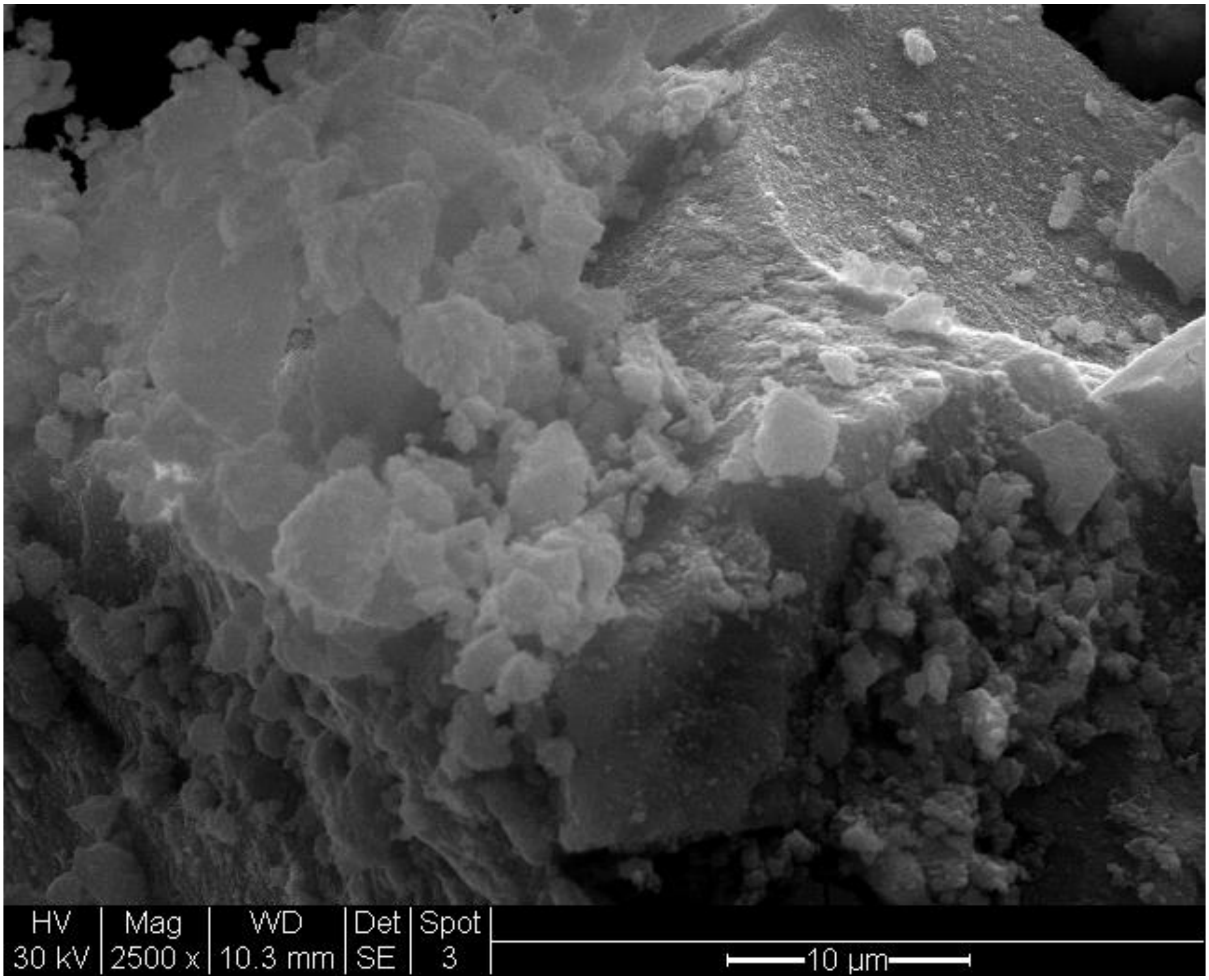

Figure S6. SEM analysis of recycled protuberant lychee-like goethite after sixth run

EDX analysis of protuberant lychee-like goethite

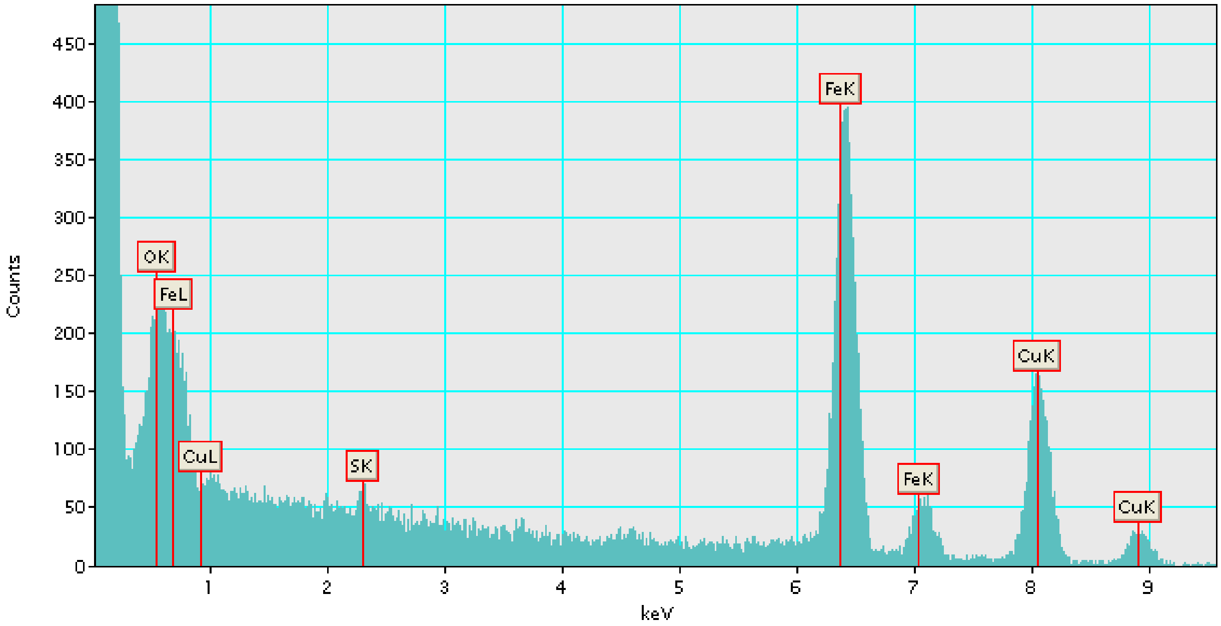

Figure S7. EDX analysis of protuberant lychee-like goethite

XPS analysis of protuberant lychee-like goethite

X-ray photoelectron spectroscopy (XPS) was used to confirm the presence of Fe (III) in protuberant lychee goethite. As shown in Figure S4, the photoelectron peaks at 711 eV (Fe $2p_{3/2}$) and 726 eV (Fe $2p_{1/2}$) of protuberant goethite show the formation of Fe^{+3}.

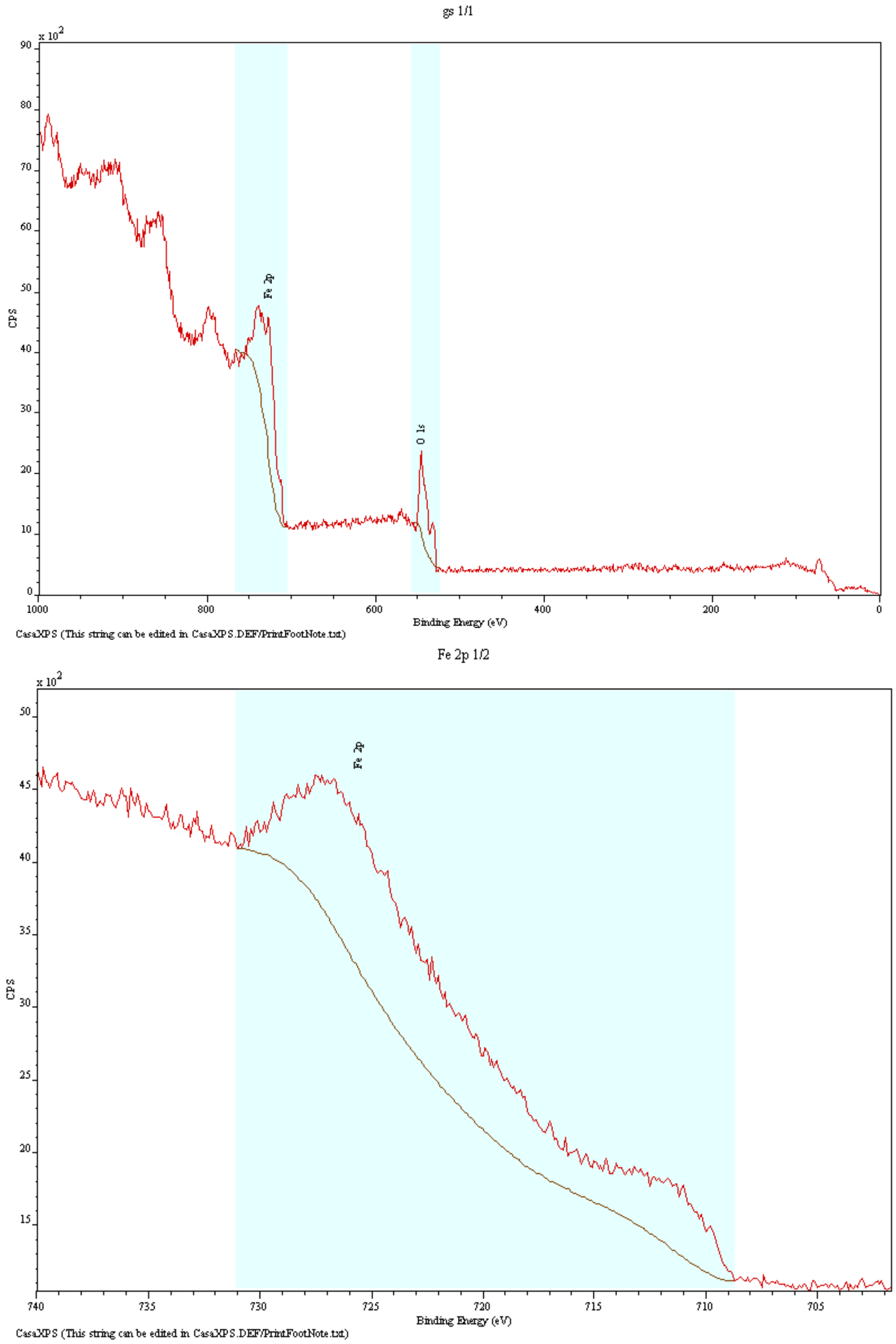

Figure S8. XPS analysis of PLG

^{1}HNMR and ^{13}CNMR of the products

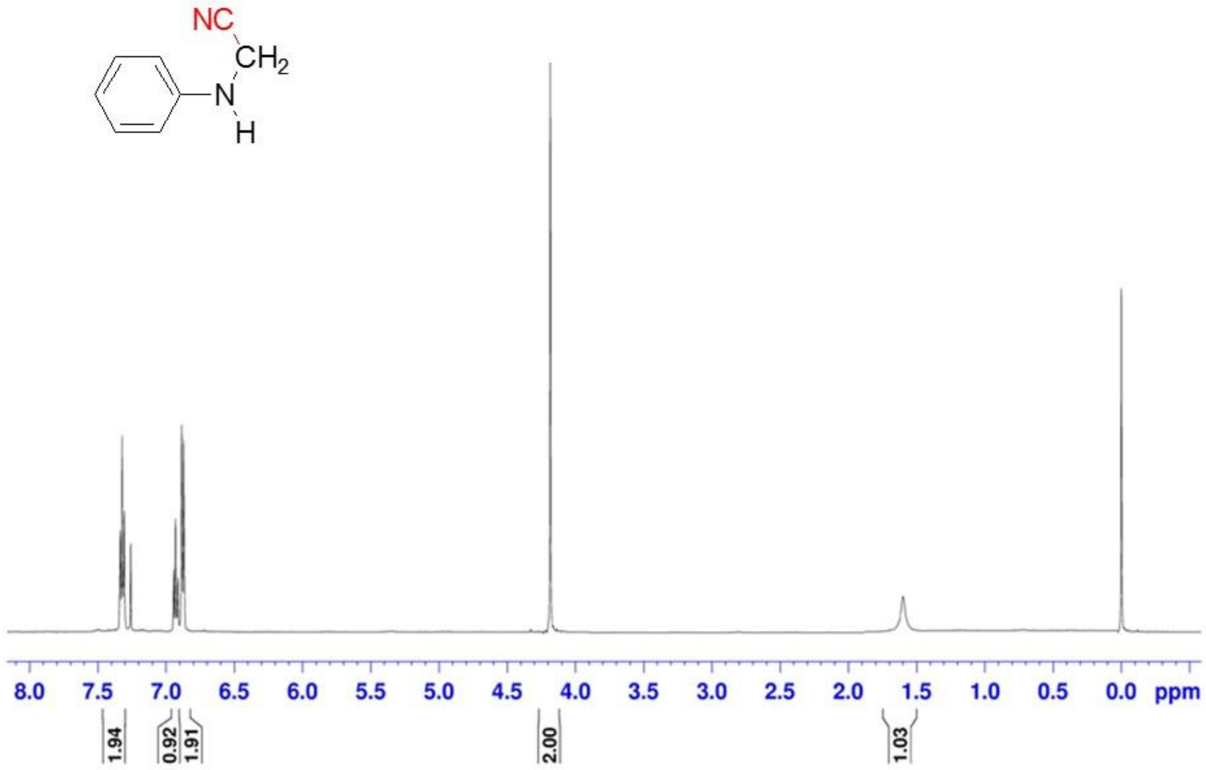

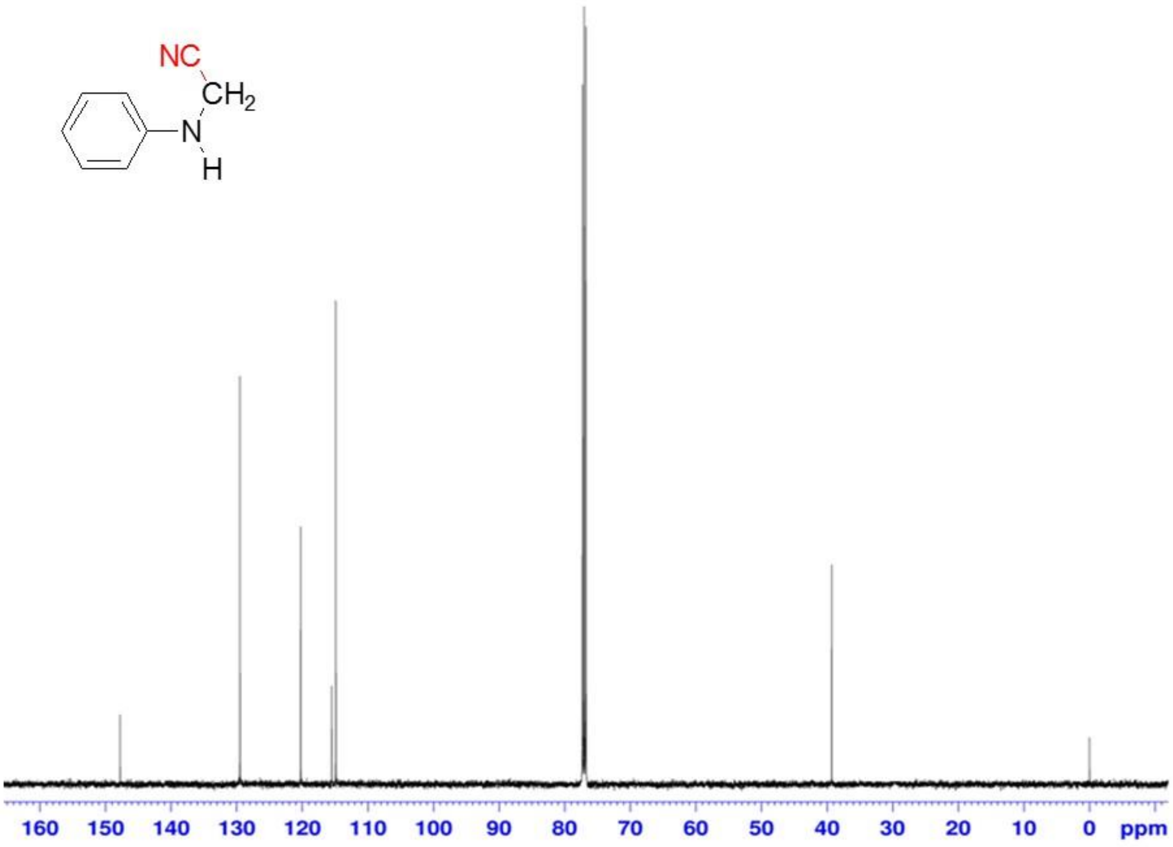
NC
CH2
N
H
160 150 140 130 120 110 100 90 80 70 60 50 40 30 20 10 0 ppm

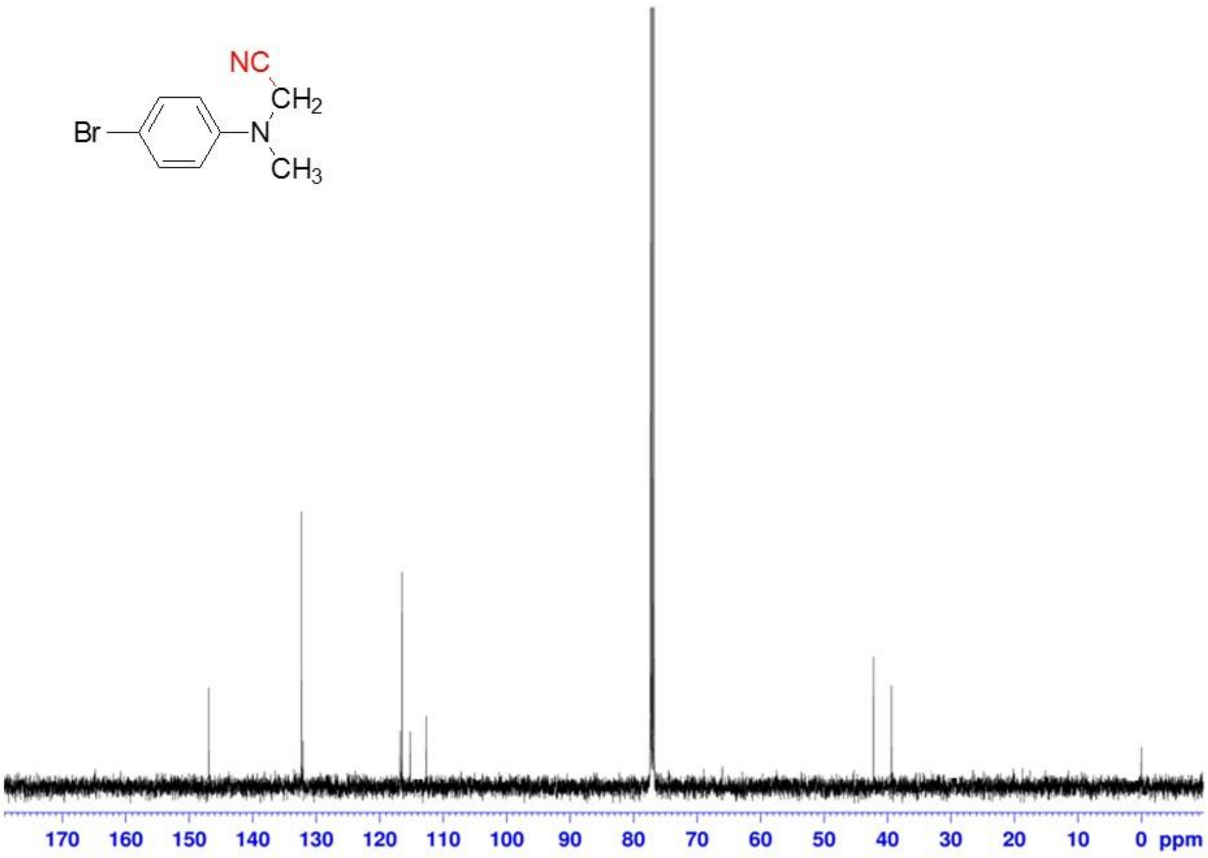
NC
CH2
Br
N
CH3
170 160 150 140 130 120 110 100 90 80 70 60 50 40 30 20 10 0 ppm

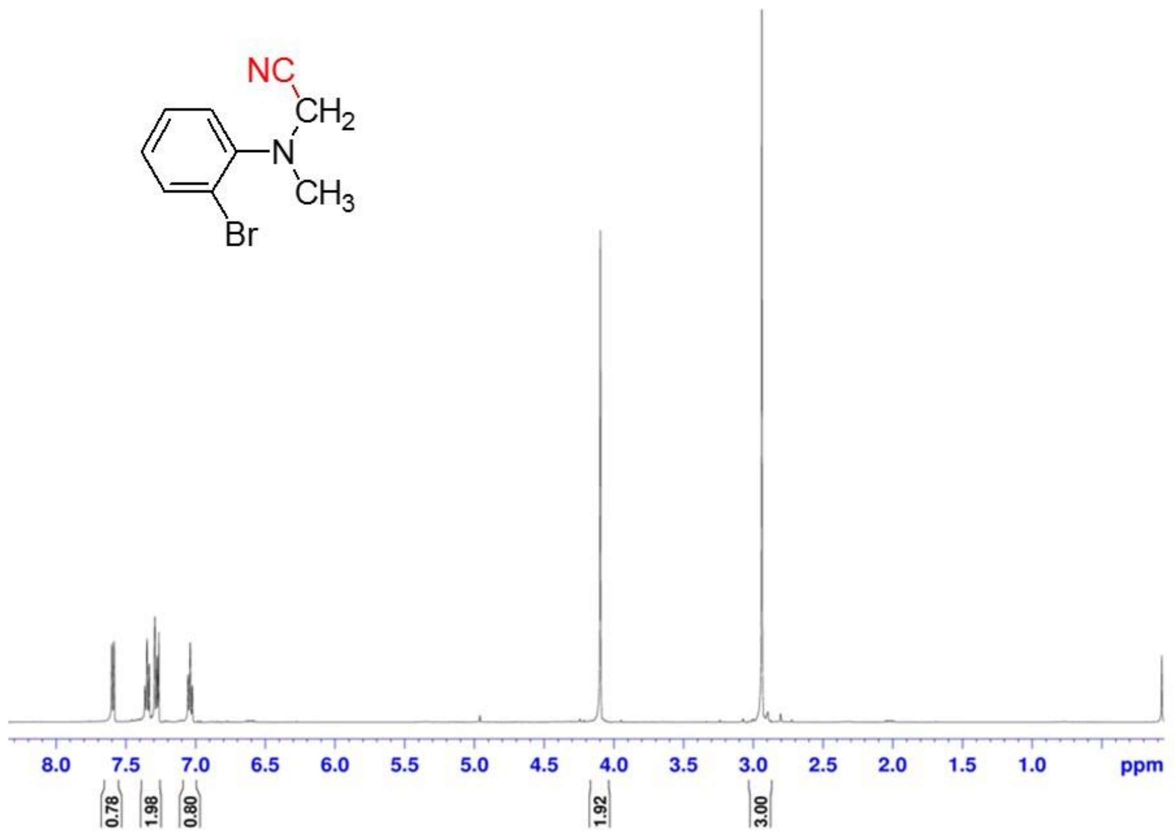

NC
CH2
N
CH3
Br
8.0
7.5
7.0
6.5
6.0
5.5
5.0
4.5
4.0
3.5
3.0
2.5
2.0
1.5
1.0
ppm
0.78
1.98
0.80
1.92
3.00

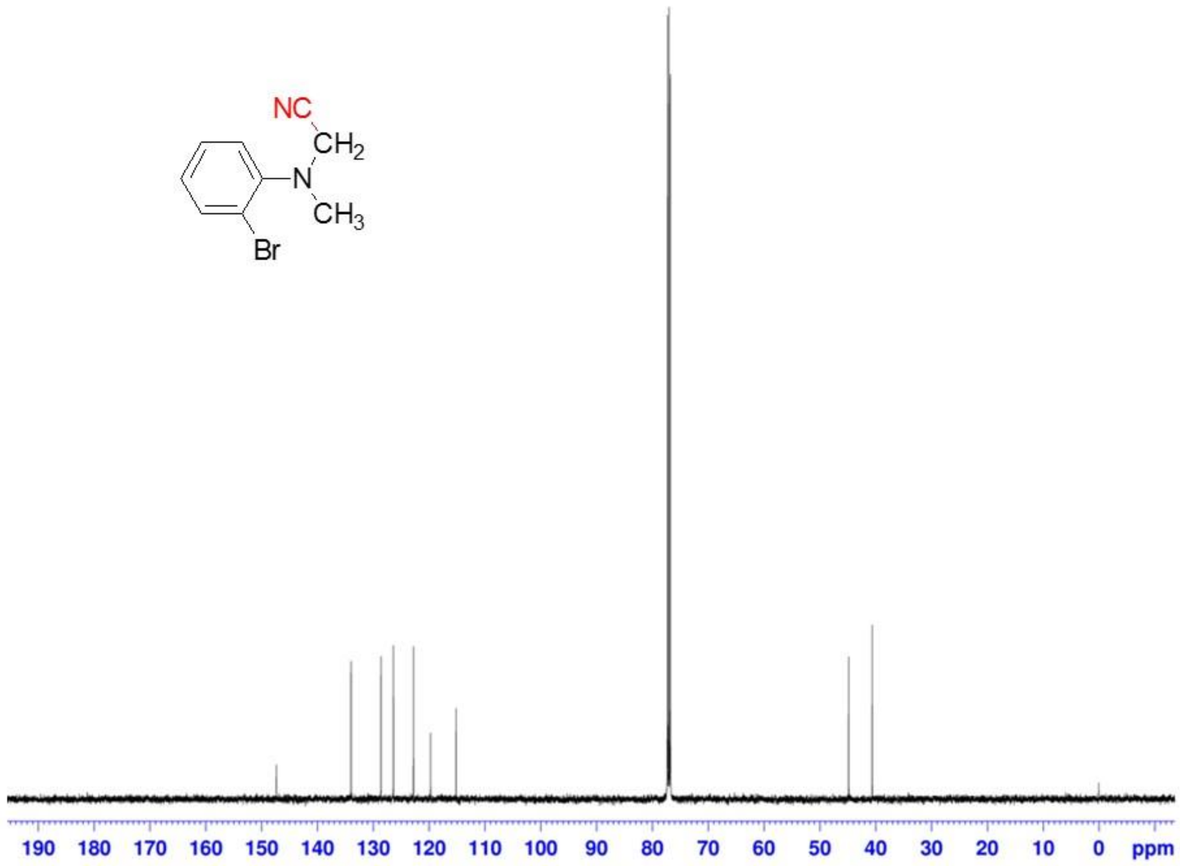

NC
CH2
N
CH3
Br
190 180 170 160 150 140 130 120 110 100 90 80 70 60 50 40 30 20 10 0 ppm

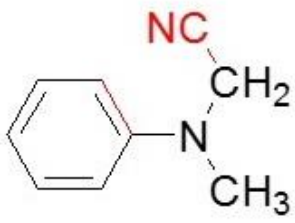
NC
CH2
N
CH3

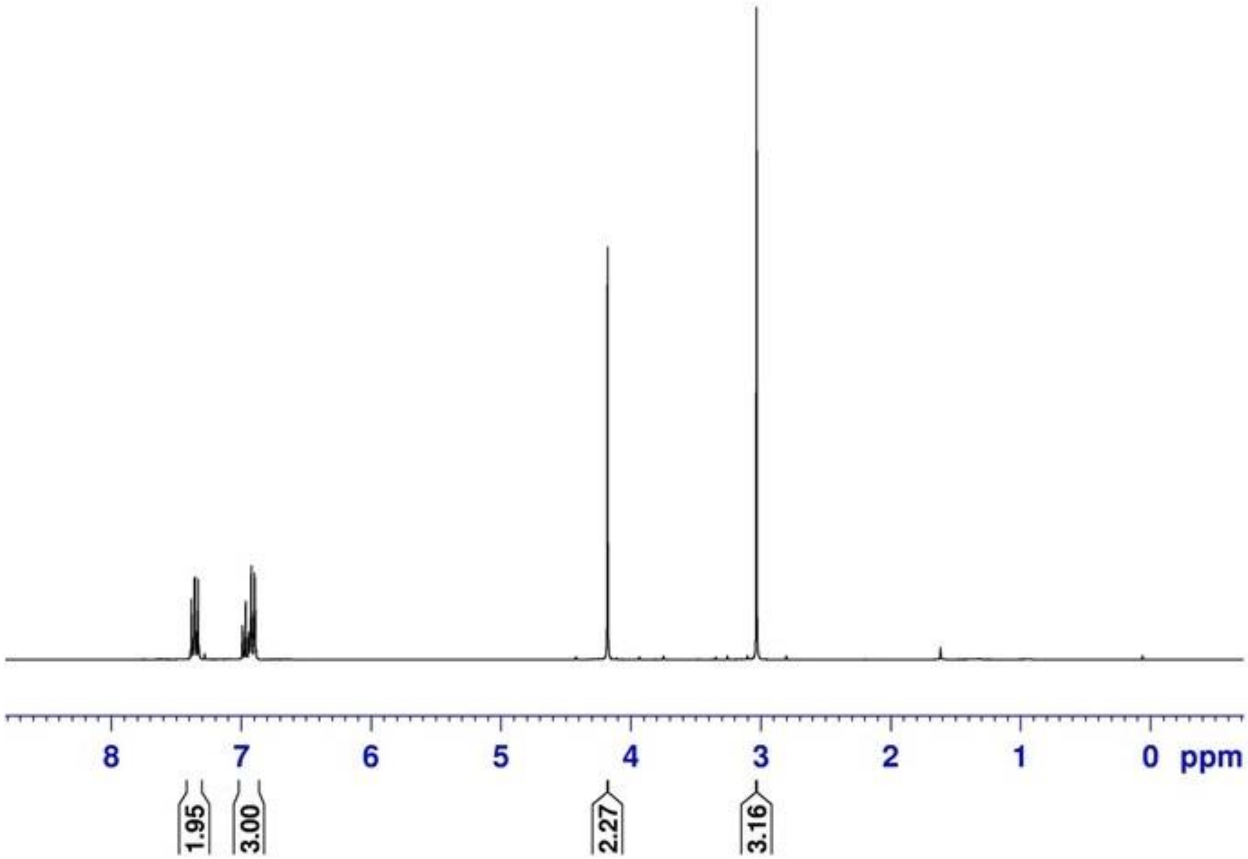
8
7
6
5
4
3
2
1
0 ppm
1.95
3.00
2.27
3.16

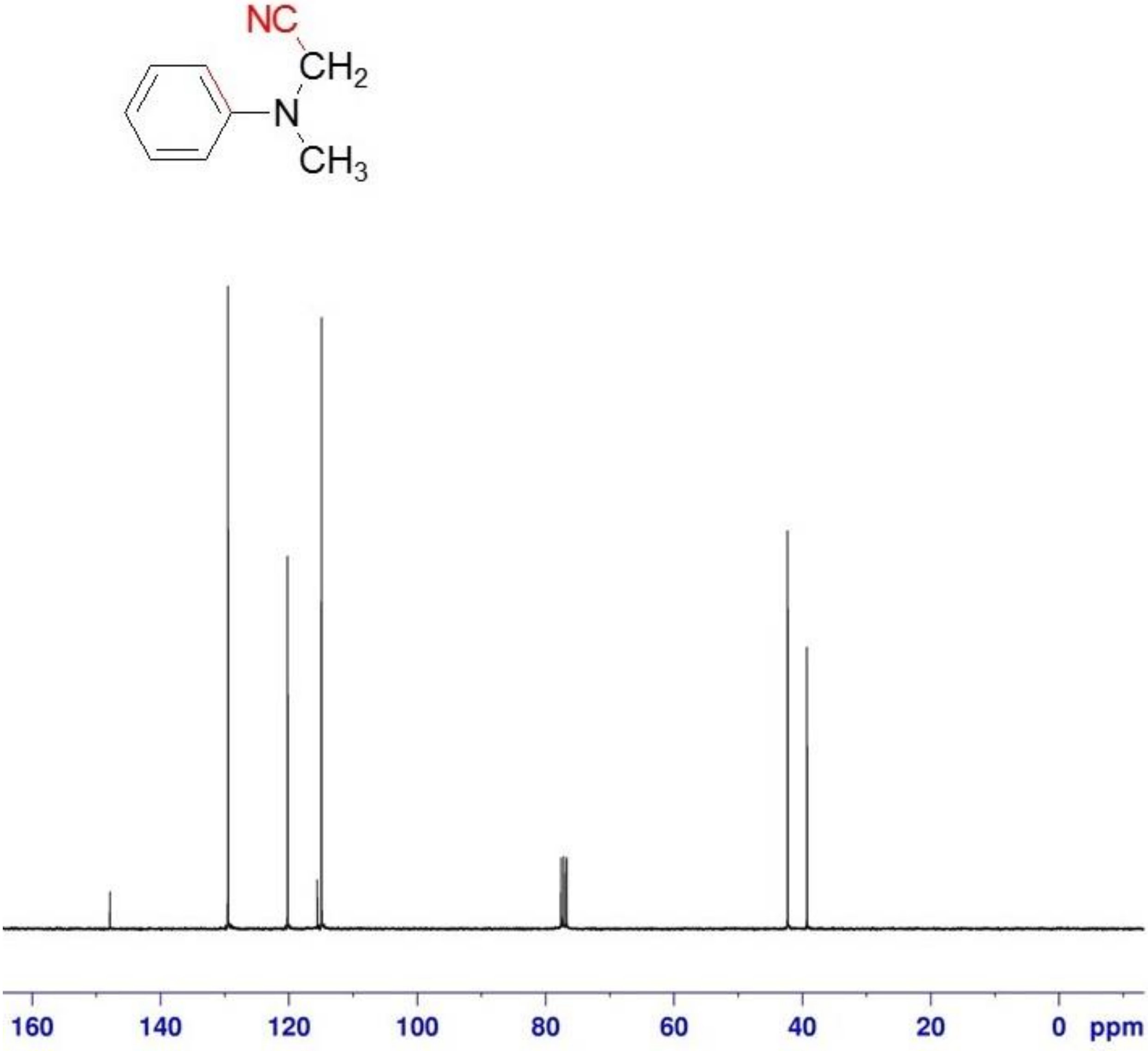
NC
CH2
N
CH3
160
140
120
100
80
60
40
20
0
ppm

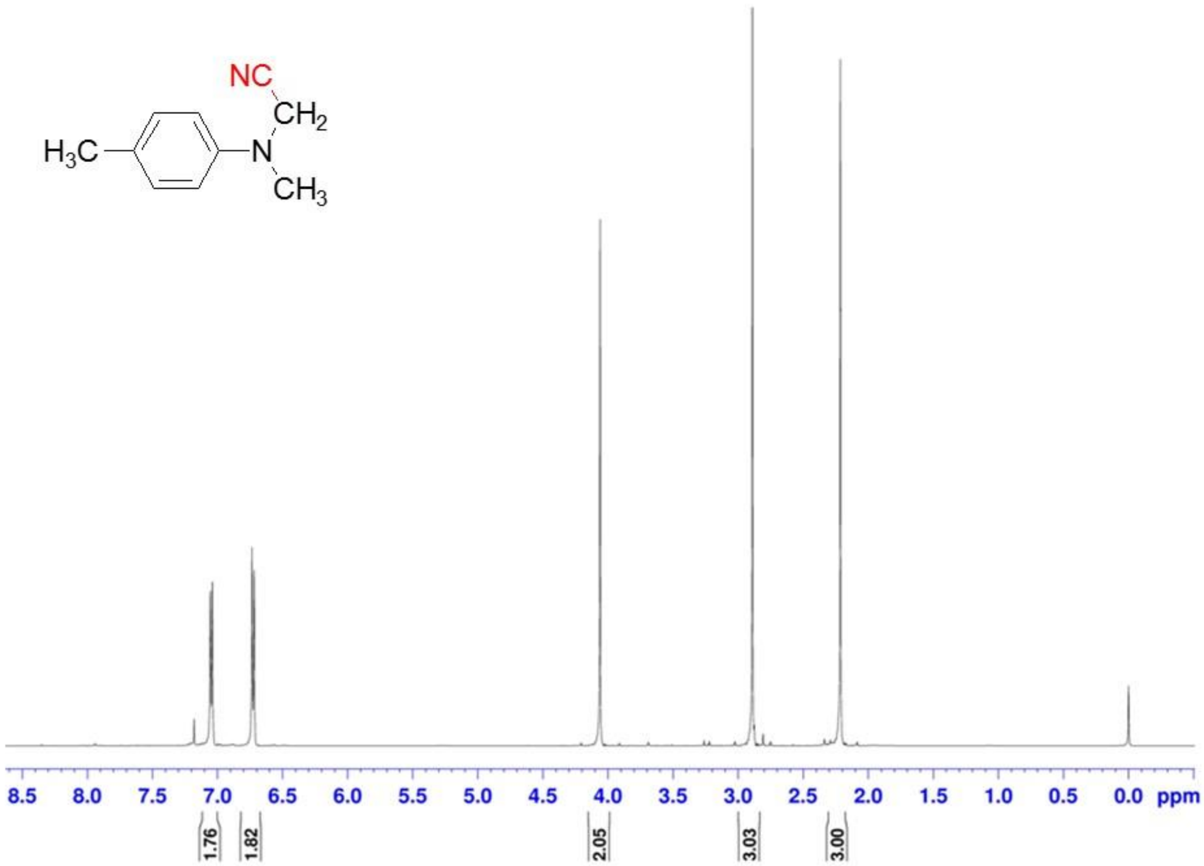
NC
CH2
H3C
N
CH3
8.5 8.0 7.5 7.0 6.5 6.0 5.5 5.0 4.5 4.0 3.5 3.0 2.5 2.0 1.5 1.0 0.5 0.0 ppm
1.76
1.82
2.05
3.03
3.00

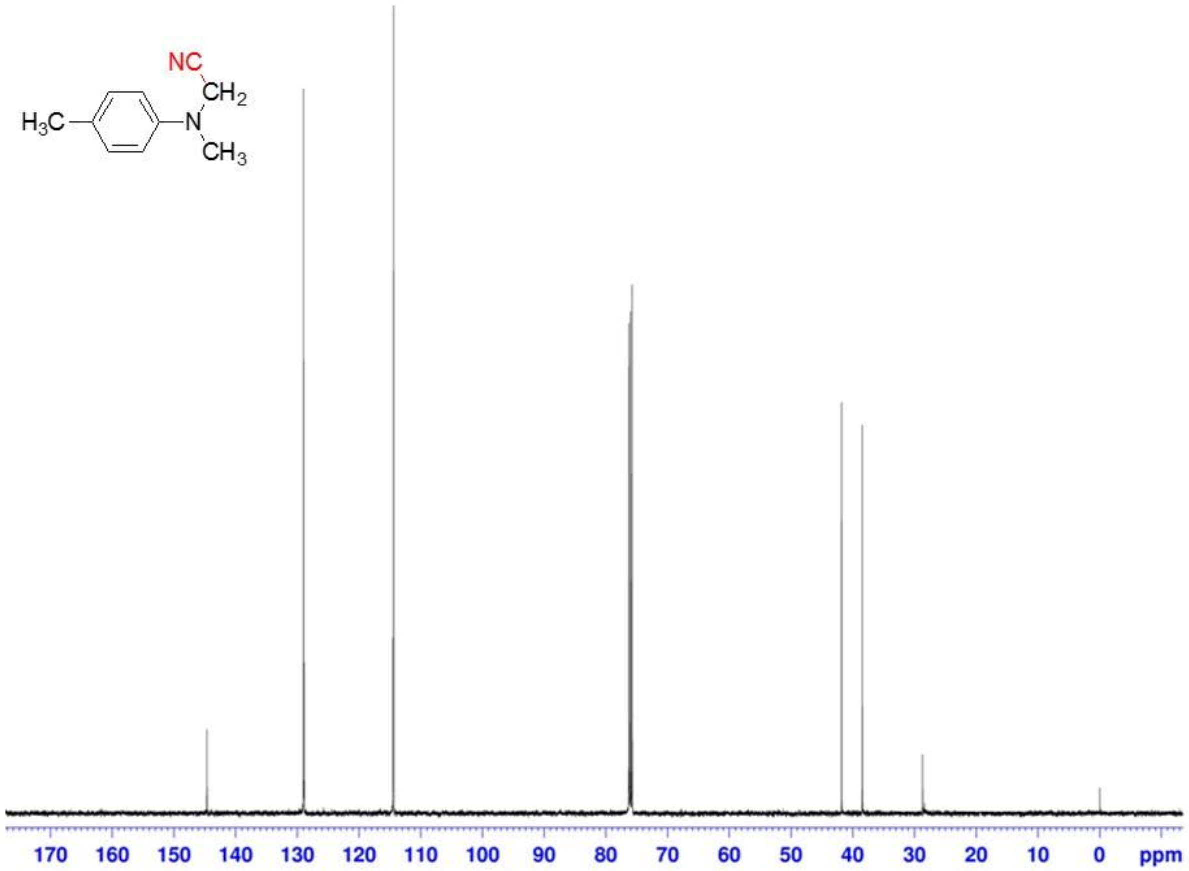
NC
CH2
H3C
N
CH3
170 160 150 140 130 120 110 100 90 80 70 60 50 40 30 20 10 0 ppm

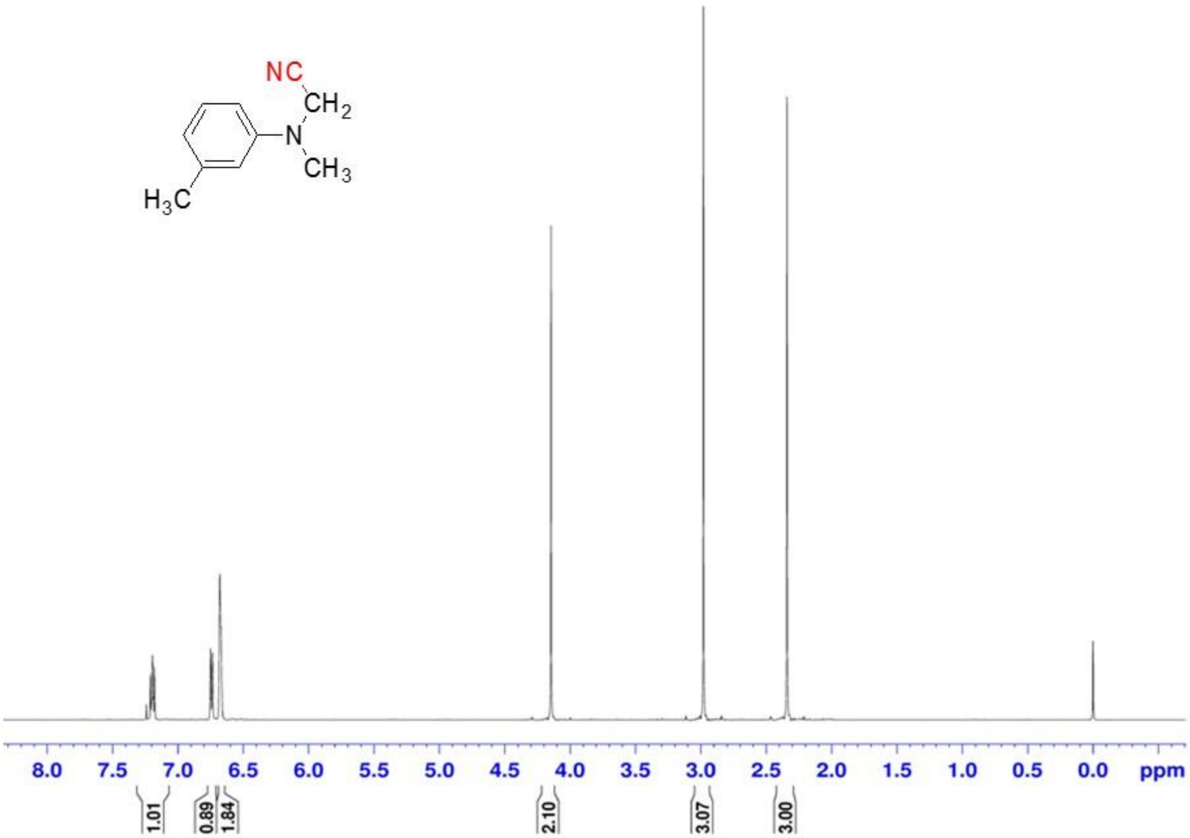

NC
CH2
N
CH3
H3C
8.0 7.5 7.0 6.5 6.0 5.5 5.0 4.5 4.0 3.5 3.0 2.5 2.0 1.5 1.0 0.5 0.0 ppm
1.01
0.89
1.84
2.10
3.07
3.00

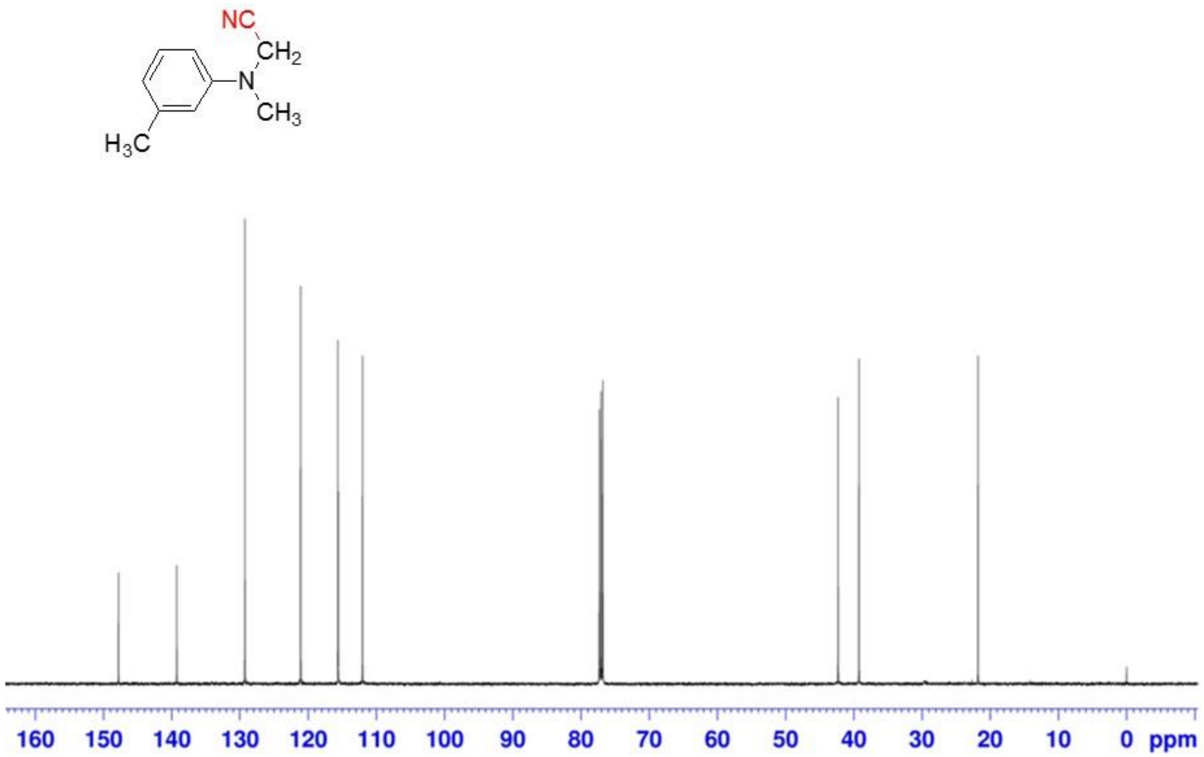
NC
CH2
N
CH3
H3C
160 150 140 130 120 110 100 90 80 70 60 50 40 30 20 10 0 ppm
